# Agomelatine for the treatment of generalized anxiety disorder: focus on its distinctive mechanism of action

**DOI:** 10.1177/20451253221105128

**Published:** 2022-06-30

**Authors:** Mark J. Millan

**Affiliations:** Institute of Neuroscience and Psychology, College of Medicine, Vet and Life Sciences, Glasgow University, 28 Hillhead Street, Glasgow G12 8QB, UK

**Keywords:** 5-HT_2C_ receptor, fear, GABA, glutamate, melatonin receptor, stress

## Abstract

Generalized anxiety disorder (GAD), the most frequently diagnosed form of anxiety, is usually treated by cognitive-behavioural approaches or medication; in particular, benzodiazepines (acutely) and serotonin or serotonin/noradrenaline reuptake inhibitors (long term). Efficacy, compliance, and acceptability are, however, far from ideal, reinforcing interest in alternative options. Agomelatine, clinically employed in the treatment of major depression, expresses anxiolytic properties in rodents and was effective in the treatment of GAD (including severely ill patients) in several double-blind, short-term (12 weeks) and relapse-prevention (6 months) studies. At active doses, the incidence of adverse effects was no higher than for placebo. Agomelatine possesses a unique binding profile, behaving as a melatonin (MT_1_/MT_2_) receptor agonist and 5-HT_2C_ receptor antagonist, yet recognizing neither monoamine transporters nor GABA_A_ receptors. Extensive evidence supports a role for 5-HT_2C_ receptors in the induction of anxious states, and their blockade likely plays a primary role in mediating the anxiolytic actions of agomelatine, including populations in the amygdala and bed nucleus of stria terminalis, as well as the hippocampus. Recruitment of MT receptors in the suprachiasmatic nucleus, thalamic reticular nucleus, and hippocampus appears to fulfil a complimentary role. Downstream of 5-HT_2C_ and MT receptors, modulation of stress-sensitive glutamatergic circuits and altered release of the anxiogenic neuropeptides, corticotrophin-releasing factor, and vasopressin, may be implicated in the actions of agomelatine. To summarize, agomelatine exerts its anxiolytic actions by mechanisms clearly distinct from those of other agents currently employed for the management of GAD.

**Plain Language Summary:**

**How agomelatine helps in the treatment of anxiety disorders**

**Introduction:**

• Anxiety disorders have a significant negative impact on quality of life.

• The most common type of anxiety disorder, called generalized anxiety disorder (GAD), is associated with nervousness and excessive worry.

• These symptoms can lead to additional symptoms like tiredness, sleeplessness, irritability, and poor attention.

• GAD is generally treated through either cognitive-behavioural therapy or medication. However, widely used drugs like benzodiazepines and serotonin reuptake inhibitors have adverse effects.

• Agomelatine, a well-established antidepressant drug, has shown anxiety-lowering (‘anxiolytic’) properties in rats and has been shown to effectively treat GAD with minimal side effects.

• However, exactly how it acts on the brain to manage GAD is not yet clear.

• Thus, this review aims to shed light on agomelatine’s mechanism of action in treating GAD.

**Methods:**

• The authors reviewed studies on how agomelatine treats anxiety in animals.

• They also looked at clinical studies on the effects of agomelatine in people with GAD.

**Results:**

• The study showed that agomelatine ‘blocks’ a receptor in nerve cells, which plays a role in causing anxiety, called the 5-HT_2C_ receptor.

• Blocking this receptor, especially in specific brain regions such as nerve cells of the amygdala, bed nucleus of stria terminalis, and hippocampus, produced the anxiety reduction seen during agomelatine treatment.

• Agomelatine also activates the melatonin (MT) receptor, which is known to keep anxiety in check, promote sleep, and maintain the sleep cycle.

• Agomelatine should thus tackle sleep disturbances commonly seen in patients with GAD.

• Beyond 5-HT_2C_ and MT receptors, signalling molecules in nerve cells that are known to be involved in anxiety disorders (called ‘neurotransmitters’ and ‘neuropeptides’) are also affected by agomelatine.

**Conclusion:**

• Agomelatine’s anxiolytic effects are caused by mechanisms that are distinct from those of other medications currently used to treat GAD.

• This explains its therapeutic success and minimal adverse side effects.

**Figure:**
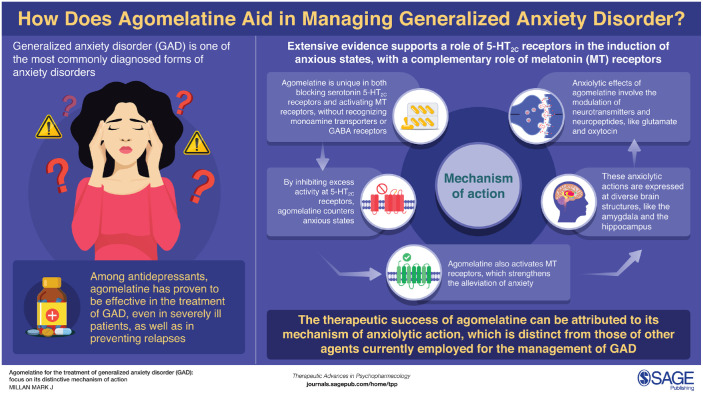
Infographic

**Figure:**
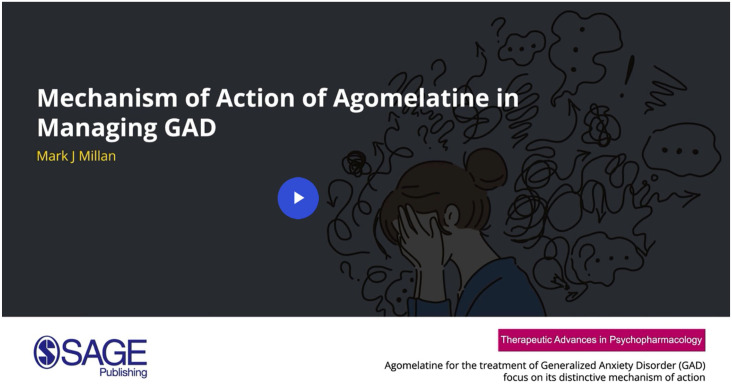
**Video Abstract** Please click on the image to play the video (also available as supplemental material).

## Introduction: core features of GAD and its current treatment

Anxiety disorders are a diverse assemblage of conditions that have a serious and negative impact on quality of life. When diagnosed in children, adolescents, and young adults, they often persist into later life. The most common class of anxiety disorder is GAD, which unfortunately is becoming more prominent amid the current COVID pandemic.^1^ Generalized anxiety disorder (GAD) is typically characterized by pervasive anxiety and nervousness, disproportionate worry, and over-generalization of genuine fear to neutral or ambivalent stimuli, sometimes on the basis of previous adverse experiences.^2–6^ Distressing emotions and thoughts are difficult to control, persist over months, and are associated with symptoms like fatigue, insomnia, irritability, poor concentration, attentional deficits, and physical complaints. Accordingly, GAD disrupts social and familial relationships and interferes with work and daily activities. Furthermore, GAD is frequently comorbid with other anxious states like social phobia and also with dysthymia or frank depression.^2,5,7^

Among a range of potential therapies, benzodiazepines are usually reserved for the immediate and acute (hours to days) control of GAD owing to the risk of dependence and a withdrawal syndrome, in addition to sedation and impairment of cognition.^2^ First-line and long-term treatment is mainly oriented around cognitive-behavioural and relaxation techniques, as well as the administration of selective serotonin reuptake inhibitors (SSRIs) and serotonin/noradrenaline reuptake inhibitors (SNRIs).^2,8–14^ In certain (rare) cases, the 5-HT1A partial agonist, buspirone, is prescribed.^2,14^ Furthermore, the anti-epileptic/analgesic and gabapentoid, pregabalin, may sometimes be administered. However – especially in association with recreational drugs and in patients with substance-abuse disorders – it presents a risk of misuse and addiction, while potential, ion channel-mediated toxic actions should also not be neglected.^15–17^ Where treatment-resistance or intolerance is encountered with standard medication, other agents may be considered such as the antidepressants, imipramine, mirtazapine, and trazodone, and (usually as adjuncts and in low doses) second-generation antipsychotics like olanzapine and quetiapine.^13,18^

A broad range of agents acting *via* contrasting molecular substrates is, then, available for the control of GAD. However, they all possess disadvantages in terms of incomplete efficacy, irresponsive patients and undesirable secondary actions. For example, some patients cannot tolerate SSRIs and SNRIs, and hence do not properly comply with their prescription. These limitations underlie continuing efforts to find improved – and mechanistically distinct – medication for the treatment of GAD.^2,13,19–21^

**Figure 1.**
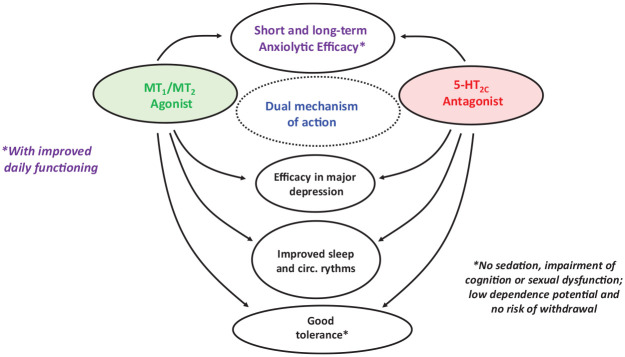
Schematic overview of the dual molecular mechanism of action of agomelatine in relation to its influence upon behaviour and its clinical properties. Agomelatine was active in several, short-term (12-week) clinical GAD trials and in a 6-month relapse-prevention study, displaying good tolerance. It is likewise effective in major depression. Based on studies in major depression and healthy subjects, Agomelatine should improve circadian rhythms and sleep patterns in GAD patients.

The present article focusses on one such agent, agomelatine (Figure 1). In the wake of early studies documenting its anxiolytic properties in rodents, clinical studies have found that agomelatine is efficacious in the treatment of GAD. Agomelatine possesses a *distinctive* binding profile/mode of action which can be related both to its therapeutic efficacy in GAD *and* to its comparatively good acceptability compared to other agents.

## Agomelatine as a novel and mechanistically distinct option for GAD

In 2009, agomelatine was launched in Europe for the treatment of major depressive episodes in adults, and it was progressively authorized for use in major depression across a suite of countries in Asia, Africa, Australasia, and South America. (At that time, the parent company Servier was not present in the United States: while now represented, the focus is on Oncology and Research). Agomelatine was the first antidepressant to be licenced that possesses a non-monoaminergic component of activity, its unique pharmacological profile comprising dual-antagonist properties at 5-HT_2C_ receptors as well as agonist properties at melatonin (MT)_1_ and MT_2_ receptors^22–25^ (see further below). This pattern of binding differs from all other classes of antidepressant currently in use, and it is distinct to the aforementioned agents clinically employed to treat GAD. Furthermore, by contrast to benzodiazepines, agomelatine does not interact with either ortho or allosteric sites on GABA_A_ receptors. In addition, agomelatine does not bind to the gabapentin-responsive alpha2delta subunit of voltage-dependent Ca^2+^ channels. In contrast to buspirone, it is devoid of affinity for 5-HT_1A_ receptors and, in distinction to SSRIs and SNRIs, agomelatine does not recognize monoamine reuptake sites.^22,23,25^

The first indications that agomelatine might be of use for the management of GAD (and anxious states in general) emerged from experimental work in rodents.^26^ Potential therapeutic efficacy in GAD was subsequently assessed within the framework of controlled clinical trials over 2008 to 2018, and these observations constitute the basis for a dossier in preparation for submission to the appropriate Health Authorities.^27,28^ These observations are consecutively summarized below and then its potential mechanisms of action are considered in greater detail.

## Anxiolytic properties of agomelatine: actions in animal models

In recent years, considerable efforts have been made to ameliorate the validity of animal models of anxiety, both for characterization of the underlying pathophysiology and for the improved detection of novel anxiolytics: in parallel, several ‘translational’ initiatives have been undertaken for the improved appraisal of potential anxiolytic activity in human subjects.^29–38^ Yet no *specific* animal model for GAD, a multidimensional and complex disorder, has to date been described.

On the contrary, an exaggerated response to fear is common in GAD patients^3,6^ suggesting that conditioned fear procedures in rats may have significant construct value for GAD. It is, thus, of note that agomelatine robustly reduced the freezing response to a conditioned aversive stimulus in rats.^39^ Agomelatine has also been evaluated in a suite of other paradigms mirroring diverse dimensions of anxious states. One example is a Vogel Conflict (approach-avoidance) procedure, whereby anxiolytic agents release a response for food or water suppressed by a mild punishment – independently of any potential influence upon appetite or nociceptive thresholds.^40^ Another example is provided by active Social Interaction with an unknown conspecific:^26,41^ this is of note because there is increasing interest in overlapping features and cellular substrates of GAD and social anxiety.^42,43^ In these and certain other procedures of potential anxiolytic properties, agomelatine displayed robust efficacy, though it has not invariably proven active in elevated plus maze and conditioned ultrasonic vocalization (USV) procedures^23,24,26,41,44,45^ (Table 1). Where active, the anxiolytic actions of agomelatine are expressed both acutely and upon sustained (several weeks) administration.^22,23,26,44^

**Table 1.** Summary of studies exploring the respective roles of 5-HT_2C_ antagonist *versus* melatonin agonist properties in the anxiolytic actions of agomelatine in rodents.

Model	Vogel conflict	Geller conflict	Social interaction	Social defeat	Plus maze
Agomelatine alone	Yes	Yes	Yes	Yes	Yes/No
5-HT_2C_ antagonists	Yes	Yes	Yes	Not tested	No
Melatonin alone	No	No	No	Yes (partial)	Yes/No
MT antagonist *vs.* agomelatine	Not blocked	Not tested	Not blocked	Not tested	Blocked

MT, melatonin.

Agomelatine was compared to several selective 5-HT_2C_ antagonists and/or to melatonin under identical conditions. The activity of agomelatine in the social defeat model was abolished by ablation of the MT_1_ receptor–rich suprachiasmatic nucleus. Variable results have been acquired with both agomelatine and melatonin in the elevated plus maze. In one study where agomelatine was effective, its actions were blunted by administration of the melatonin antagonist, S22153. This drug was likewise employed in interaction with agomelatine in the Vogel conflict and Social interaction procedures where it was, by contrast, inactive. For details, see main text.

Interestingly, agomelatine also counters anxiety-related behaviours in several rodent models of ‘depression’, including pre-natal or chronic stress.^23,46,47^ These observations, together with its clinically proven antidepressant properties,^22^ support the use of agomelatine for helping patients with mixed anxious-depressive states^7,48^ – a possibility yet to be formally addressed in dedicated clinical trials.

## Anxiolytic properties of agomelatine: actions in clinical studies of GAD

In the wake of the encouraging experimental findings outlined above, clinical efficacy of agomelatine (25–50 mg/day) was evaluated in patients suffering from GAD. Efficacy *versus* placebo was demonstrated in three independent, double-blind, ‘short-term’ (12-week) studies that employed both the Hamilton Anxiety Scale as well as the Sheehan Disability Scale to monitor functional impairment.^49–51^ The positive outcome of these respective studies was recently reprised by a pooled meta-analysis that underpinned evidence for robust efficacy both in alleviating symptoms and in enhancing global patient function.^27,28^ Efficacy of agomelatine was comparable to the active control, escitalopram (an SSRI), and secondary analysis supported effectiveness in severe GAD (Hamilton Anxiety Scale > 21).^28,49–52^ Although its precise onset of efficacy remains to be further characterized, clinical studies suggest activity within the 1–3 weeks after commencing administration in at least some patients.^28,49–52^ A further study undertaken over 6 months demonstrated efficacy in preventing relapse.^53^

Despite concerns from depressed patients about a dose-dependent (albeit low) risk of hepatotoxicity that necessitates control of liver function,^22,54^ only a small percentage (1.8%) of patients in the short-term studies of GAD showed potentially significant increases in transaminases: there were no cases of liver disease and transaminase levels normalized after stopping administration in all patients. This issue obviously requires close future surveillance, but data in GAD are so far reassuring, and recent comparative analyses of agomelatine with other antidepressants in major depression reinforce this conclusion.^55–57^ Furthermore, tolerance was good in GAD patients with no difference in the frequency of discontinuation-related adverse effects in the agomelatine (headache, nasopharyngitis, and nausea) *versus* placebo groups (both 2.1%). In addition, there was no evidence for an agomelatine withdrawal syndrome in either the short-term or relapse-prevention studies.^28,49–53^ These observations are consistent with clinical observations acquired in studies of its antidepressant properties.^22,56^ More specifically, they support the notion that the distinctive receptor-binding profile of agomelatine should not be associated with the risks of tolerance, dependence/withdrawal, and recreational abuse that burden benzodiazepines. Agomelatine lacks affinity for the 5-HT transporter,^22,23^ and clinical work bears out the low risk of disrupted sexual function and sleep – or an acute exacerbation of anxiety – at the onset of treatment. This represents an important gain over SSRIs and SNRIs – and may also be an advantage compared with buspirone.^13,22,23,27,28,57–62^

Activation of 5-HT_2C_ receptors, for example, on hypothalamic proopiomelanocortin neurons, suppresses appetite. Conversely, 5-HT_2C_ receptor blockade, in particular when coupled to histaminergic and/or muscarinic receptor antagonism, is a risk factor for increased food consumption, obesity, and metabolic dysregulation, as seen with numerous tricyclic antidepressants and ‘atypical’ antipsychotics like olanzapine.^63–68^ It is of note, then, that agomelatine does *not* recognize histaminergic, muscarinic, or other classes of receptor incriminated in triggering weight gain.^22,23^ In addition, agomelatine is a neutral antagonist rather than inverse agonist at 5-HT_2C_ receptors, so it is does not decrease 5-HT_2C_ receptor–mediated transmission to below ‘normal or default’ levels.^69,70^ These characteristics suggest that agomelatine has a low risk of metabolic perturbation and obesity, an assertion underscored by clinical observations in studies of both GAD and major depression.^22,28^ There is also a correspondingly low risk of rebound anxiety or a discontinuation syndrome at the end of treatment.^22,28,70,71^

To recap, then, the distinctive 5-HT_2C_ antagonist/MT agonist receptor-binding profile of agomelatine can be related both to its therapeutic efficacy in GAD and to its good tolerance.^13,27,28,58^ Its favourable clinical profile was recently underscored in two separate meta-analyses of a diversity of agents clinically evaluated for the treatment of GAD.^13,58^ Nonetheless, for a more fine-grained and complete understanding of the mechanisms of action of agomelatine in the control of GAD, it is instructive to consider a suite of observations acquired mainly in rodents.

## Anxiolytic actions of agomelatine: 5-HT_2C_ receptor blockade compared to MT agonist properties

As regards the mechanism of action of agomelatine in the expression of its anxiolytic actions, the primary focus has not surprisingly been on the respective role of 5-HT_2C_ as compared to MT receptors. Employing agomelatine-responsive anxiolytic procedures in rats, comparisons have been undertaken both to 5-HT_2C_ antagonists and to MT. In addition, interaction studies have been performed with the MT_1_/MT_2_ receptor antagonist, S22153.^23^ The key observations acquired are depicted in Table 1 and briefly outlined below.

In a Vogel conflict procedure undertaken in mildly (overnight) water-deprived rats, the ability of agomelatine to disinhibit punished (weak electric shock on the spout) was mimicked under identical conditions by several different selective 5-HT_2C_ receptor antagonists, whereas MT was inactive.^26^ Similar observations have been made employing the related Geller (mild food-deprivation) procedure.^23^ In addition, S22153 failed to block the anxiolytic actions of agomelatine in these paradigms. Comparable results were obtained in a model of active social interaction between two unfamiliar rats presented to each other in an unfamiliar (open-field) environment.^26^ These observations strongly suggest that 5-HT_2C_ receptor blockade is necessary and sufficient for the expression of anxiolytic properties in the above procedures. In a separate study, S22153 enhanced (for not entirely clear reasons) the suppressive influence of agomelatine upon USVs provoked by conditioned fear: re-exposure to an environment previously associated with an aversive stimulus. Conversely, in a study of the elevated plus maze, the anxiolytic actions of agomelatine were blunted by S22153.^41^

Taken together, these findings suggest a major role for 5-HT_2C_ receptor blockade in the anxiolytic actions of agomelatine. Supporting this assertion, its 5-HT_2C_ antagonist properties are expressed over a similar dose-range in several pharmacological models.^23^ In addition to this preponderant role for 5-HT_2C_ receptor antagonist properties, there appears to be a complementary role for MT receptor agonism in the anxiolytic profile of agomelatine. Further evidence underpinning the respective roles of 5-HT_2C_ and MT receptors is outlined in the following sections.

## Key role for 5-HT_2C_ receptors in the anxiolytic actions of agomelatine: supporting studies in rodents and humans

In the light of the above-discussed evidence that 5-HT_2C_ receptor antagonism participates in the anxiolytic actions of agomelatine, it is instructive to evoke studies undertaken in animals and in humans that underpin a role for 5-HT_2C_ receptor blockade in the relief of GAD and anxious states.

First, paralleling observations obtained in direct, side-by-side comparisons with agomelatine, diverse classes of 5-HT_2C_ receptor antagonist exert anxiolytic properties across a range of animal models. Conversely, 5-HT_2C_ receptor agonists generally display anxiogenic properties.^71–75^ Second, in line with these findings, 5-HT_2C_ receptor knockout mice display an ‘anxious’ profile, though this is only seen under certain conditions and a tendency for increased locomotor activity complicates interpretation of data.^64,76,77^ Third, indirect, 5-HT-mediated activation of 5-HT_2C_ receptors mediates the acute anxiogenic actions of SSRIs in rodents, notably in the social interaction procedure in which agomelatine is anxiolytic. Conversely, upon long-term exposure, this anxiogenic effect fades and 5-HT_2C_ receptor desensitization/down-regulation likely contributes to the long-term anxiolytic effects of SSRIs: studies of hippocampal electroencephalographic activity in rats reinforce this interpretation.^78–85^ Fourth, second-line antidepressants and antipsychotics used to treat GAD (or their major metabolite in the case of quetiapine) share antagonist properties at 5-HT_2C_ receptors.^86–90^ Finally, while no selective ligand at 5-HT_2C_ receptors has yet been authorized for the therapy of GAD, the 5-HT_2C_ antagonist ritanserin abrogates the exacerbation of anxiety in GAD patients provoked by the prototypical 5-HT_2C_ agonist, ‘mCPP’ (meta-chlorophenylpiperazine). Ritanserin also blocks the anxiogenic and other effects of mCPP in non-anxious (‘normal’) subjects.^91–93^ Thus, both experimental and clinical evidence supports a role for 5-HT_2C_ receptor antagonism in the attenuation of anxious states and the relief of GAD by agomelatine.

Before moving on to MT receptors, it should be mentioned that agomelatine displays affinity comparable to that for 5-HT_2C_ receptors at closely related 5-HT_2B_ receptors: mCPP and ritanserin also interact with 5-HT_2B_ receptors.^23,63,94,95^ In contrast to 5-HT_2C_ receptors, however, there is no evidence from either pharmacological or gene knockout studies that 5-HT_2B_ receptor activation elicits anxious states, nor that their inactivation is associated with anxiolytic properties. Indeed, as compared to 5-HT_2C_ receptors, several studies have reported that 5-HT_2B_ agonists rather than antagonists display anxiolytic actions.^48,70,96,97^ Accordingly, there is no evidence for a role of 5-HT_2B_ blockade in the influence of agomelatine upon anxiety, and the discussion below focusses on 5-HT_2C_ receptors.

## A complementary role for MT receptors in the anxiolytic actions of agomelatine: supporting studies in rodents and humans

As regards a complementary role for melatonergic agonism in the anxiolytic actions of agomelatine, supporting data are less broad-based than those for 5-HT_2C_ receptor blockade. Nonetheless, a few studies have reported anxiolytic actions of MT (as a function of the procedure and time of light cycle) in rodent models like the elevated plus maze and novelty suppressed feeding tests.^41,98–103^ Most pertinently, in a paradigm of social defeat, agomelatine abrogated associated anxiety-related behaviours, and its actions were partially reproduced by MT (5-HT_2C_ antagonists were not unfortunately tested) and abolished by lesions of the MT_1_ receptor–rich suprachiasmatic nucleus (SCN).^45^ Furthermore, increases in anxiety have been documented in MT_1_ knock mice.^104^ As regards MT_2_ receptors, the synthetic MT_2_ agonist (UCM765) has been reported to mimic the anxiolytic properties of MT, and its actions were blocked by a selective MT_2_ receptor antagonist.^102^ In line with this work, male and/or female mice genetically deprived of MT_2_ receptors display enhanced anxiety.^105–108^ As regards human subjects, data are very sparse, yet there is fragmentary evidence for anxiolytic effects of MT under specific conditions, such as pre-operative stress.^100,109^

Independently of any direct influence of MT agonism on circuits mediating and controlling anxiety, MT receptor stimulation by agomelatine should be linked to an improvement (advanced onset) of sleep and circadian rhythms.^110,111^ Since sleep is commonly perturbed in patients with GAD, this would be expected to favour the relief of anxious states.^5,7,35^

## Cerebral loci of action of agomelatine in relation to fear-anxiety integrating circuits

**Figure 2.**
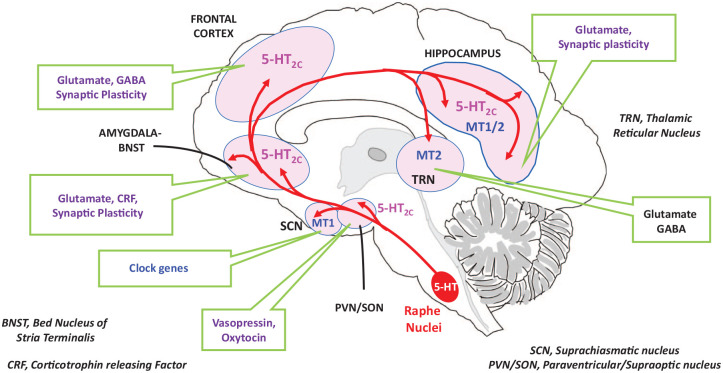
Schematic illustration of cerebral structures and neuromediators potentially involved in the anxiolytic actions of agomelatine in relation to the localization of 5-HT_2C_ and MT_1_ receptors. Several interconnected structures integrating fear and anxiety are shown. Both the thalamic reticular nucleus (TRN)–*via* the mediodorsal thalamus–as well as the ventral hippocampus project to the frontal cortex (FCX). The FCX itself innervates the bed nucleus of the stria terminalis (BNST) and the amygdala, the latter of which also projects to the BNST (Adhikari, 2014). The interlinked amygdala and BNST represent key sites for the expression of fear and anxiety. 5-HT_2C_ and MT_1_ or MT_2_ receptors in these regions are likely sites for anxiolytic actions of agomelatine, which is thought to act *via* the neural mechanisms indicated. Other potential sites of action for agomelatine include the habenula, the dorsal striatum and the periaqueductal grey (not shown, see main text). Agomelatine may also act at MT_1_ sites in the SCN (directly or *via* modulation of circadian rhythms) to affect anxious states, and *via* modulation of the secretion of vasopressin/oxytocin from paraventricular/supra-optic nuclei, likely downstream of 5-HT_2C_ receptors. This figure represents a work in progress: further study is needed to more precisely determine the cellular and neural mechanisms of anxiolytic action of agomelatine in GAD.

The above observations focussed on the significance of the primary molecular targets of agomelatine, 5-HT_2C_ and MT receptors, in the expression of its anxiolytic properties. Two interrelated questions arise. First, regarding the cerebral location of the respective populations of receptor involved and, second, concerning the roles of various downstream neurotransmitters and neuromodulators in mediating the 5-HT_2C_/MT receptor–triggered actions of agomelatine. Figure 2 presents an overview of our current knowledge in this respect which serves as a framework for the discussion below, and for future work.

Comparatively, few studies have to date been undertaken with agomelatine to specifically identify its anxiolytic loci of action in the brain. One pragmatic reason for this is the highly lipophilic nature of agomelatine, leading to rapid diffusion through neural tissue: this renders intracerebral microinjection studies problematic. Nonetheless, a functional magnetic resonance imaging (fMRI) study in rats found that agomelatine blocked the ‘BOLD’ response to a selective 5-HT_2C_ agonist (RO-60,0175) in the mediodorsal thalamus as well as the cortex, ventral hippocampus and periaqueductal grey,^112^ key structures involved in the processing of fear and anxiety in animals and humans.^6,35,113,114^ These findings support a role for 5-HT_2C_ receptors in the hippocampus in the anxiolytic actions of agomelatine. Interestingly, systemic administration of agomelatine exerts a marked influence on synaptic plasticity, diverse intracellular signals, and neuromodulators like neural cell adhesion molecule, an emotion-regulating growth factor, in this structure.^23,115,116^ Findings with selective 5-HT_2C_ receptor agonists and antagonists underscore a role of the hippocampus in the modulation of anxiety, and they also provide evidence for roles of 5-HT_2C_ receptors in the amygdala (basolateral and central nuclei) and the interconnected bed nucleus of the stria terminalis (BNST). For example, activation of 5-HT_2C_ receptors in the basolateral amygdala underlies the induction of anxiety by stimulation of the raphe nucleus.^117^ These regions comprise core elements of a stress-sensitive, fear-integrating circuit involved in the induction of anxious states that is modulated by 5-HT_2C_ receptors and, *ipso facto*, one may assume agomelatine^48,63,64,84,118–121^ (see also next section).

Like 5-HT_2C_ receptors, both MT_1_ and MT_2_ receptors are localized in the hippocampus (mainly dental gyrus and CA3 regions, respectively). The former are also highly concentrated in the SCN, whereas the latter are prominent in the thalamic reticular nucleus (TRN).^122^ As mentioned above, the approach of discrete brain lesions suggests that the integrity of the MT_1_ receptor-enriched SCN, the circadian master regulator, is required for alleviation by agomelatine of anxious behaviour following social defeat.^45^ Agomelatine interacts with circadian-rhythm-related genes (like ‘Period-1’) in the SCN (and hippocampus): studies are undergoing to determine if and how this influence relates to specific classes of anxiety disorders.^123,124^ On the contrary, the above-mentioned MT_2_ receptor agonist UCM765 activates neurons in the TRN that project *via* the dorsal medial thalamus to the frontal cortex (FCX), which itself feeds into the amygdala-BNST axis to control anxious states. Accordingly, it has been proposed that activation of MT_2_ receptors in the TRN acts *via* this neural cascade to counter anxiety, and they are a potential substrate for the anxiolytic actions of agomelatine.^102,108,125^ This possibility is especially interesting bearing in mind the role of the TRN in sleep^126^ and evidence that agomelatine influences the activity of neurons in the dorsomedial thalamus and FCX downstream of the TRN.^23,112^ Finally, a role of either MT_1_ and/or MT_2_ receptors in the hippocampus may, by analogy to their 5-HT_2C_ counterparts, be involved in the response to stress and the anxiolytic actions of MT together with, by extrapolation, agomelatine.^23,100,103,127^

## Potential neurochemical substrates of action involved in the anxiolytic actions of agomelatine

As regards neurochemical substrates involved in the anxiolytic properties of agomelatine, it is interesting to consider potential roles for glutamate and several different classes of neuropeptide.

Both 5-HT_2C_ receptor ligands and SSRIs have been found to impact stress-sensitive glutamatergic transmission in structures like the FCX, hippocampus, and amygdala.^128,129^ As regards agomelatine itself, its acute administration blunted stress-induced release of glutamate in the basolateral and central amygdala as well as the hippocampus. In the past, the tendency has been to automatically relate this modulation of glutamatergic pathways (and other neurochemical effects of agomelatine) to its antidepressant actions. However, these effects might more compellingly be interlinked with its anxiolytic properties in view of the pivotal role of the amygdala and hippocampus in the regulation of fear and anxiety.^35,46–48,113,114^

Modulation of the activity of the anxiogenic peptide, corticotrophin-releasing factor (CRF) in the amygdala and the BNST^35,130,131^ has been implicated in the influence of 5-HT_2C_ receptors – and, by extension, agomelatine – upon anxious states.^35,82,117,121,132^ Of particular interest, serotonergic pathways projecting to the BNST from the dorsal raphe act *via* 5-HT_2C_ receptors to engage a CRF circuit that inhibits the anxiolytic influence of a BNST projection to the lateral hypothalamus and ventrotegmental area. Activation of these 5-HT_2C_ receptors by SSRIs is thought to underlie their aversive/anxiogenic effects at the onset of treatment. Agomelatine would act oppositely to SSRIs in blocking BNST-located 5-HT_2C_ sites and moderating CRF output, contributing to the expression of its anxiolytic properties in the *absence* of an early phase of aggravated anxiety.^2,13,28,121^ CRF may not be the only neuropeptide potentially implicated in the actions of agomelatine. Post-weaning isolation in rats is associated with heightened anxiety in adults, together with reduced plasma levels of oxytocin (which possesses anxiolytic properties) and elevated levels of vasopressin (‘anxiogenic’).^35^ Sub-chronic (2 weeks) administration of agomelatine moderated anxiety as well as reversing the increases in vasopressin levels, and (albeit only in females) it also attenuated the fall in levels of oxytocin.^33^ These effects were specific since, despite the above-described influence on CRF in the BNST, there was no apparent influence on corticosterone levels downstream of the hypothalamic–pituitary–adrenal axis.^133^ Intriguingly, there is evidence that 5-HT_2C_ receptors physically associate with and blunt signalling at oxytocin receptors and that oxytocin hypoactivity is countered by 5-HT_2C_ antagonists including, at least in theory, agomelatine.^134^

**Figure 3.**
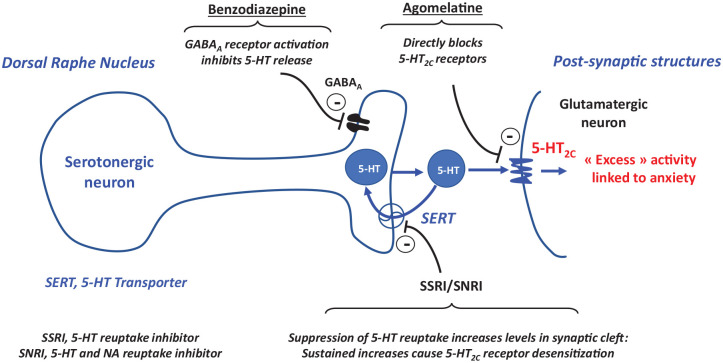
Schematic depiction of the influence of agomelatine compared to several other classes of anxiolytic upon serotonergic/5-HT_2C_ receptor-mediated neurotransmission. A prototypical serotonergic neuron is shown, projecting onto a post-synaptic glutamatergic neuron in, for example, the hippocampus. Over-activation of 5-HT_2C_ receptors contributes to anxious states. Benzodiazepine’s recruit GABA_A_ receptors to reduce the release of 5-HT, yet these GABA_A_ receptors rapidly desensitize. Agents that block the reuptake of 5-HT by terminal-localized transporters (SERT) initially increase synaptic levels of 5-HT to stimulate 5-HT_2C_ receptors: upon long-term administration, in the face of sustained and high levels of 5-HT, 5-HT_2C_ receptors desensitize. agomelatine, by contrast, directly blocks 5-HT_2C_ receptors in both the short and long term.

Serotonergic projections are subject to the inhibitory control of GABAergic interneurons expressed both at the level of terminals and of cell bodies in raphe nuclei. Accordingly, benzodiazepines suppress (‘excess’) release of 5-HT by activation of GABA_A_ receptors presynaptic to serotonergic neurons in the dorsal raphe nucleus, hippocampus, amygdala, and other regions, actions that contribute to their anxiolytic properties.^2,8,26,35,78,135^ Interestingly, at least in rodents, 5-HT_2C_ receptors are expressed by raphe-localized GABAergic interneurons targeting serotonergic pathways projecting to the basolateral amygdala.^135^ Under conditions of acute stress, 5-HT_2C_ agonists attenuate the activity of ascending serotonergic pathways^136,137^ This action, and some – albeit inconsistent – evidence for anxiolytic properties of 5-HT_2C_ agonists, likely reflect recruitment of GABAergic interneurons upstream of serotonergic pathways.^26,35,138,139^ Nonetheless, presumably reflecting the low tonic activity of 5-HT_2C_ receptors on GABAergic neurons, as assessed by dialysis in freely moving rats and at anxiolytic doses, agomelatine did *not* modify extracellular levels of 5-HT in the hippocampus or other structures^5,26^ (Figure 3). This *lack* of impact on extracellular levels of 5-HT mimics selective 5-HT_2C_ antagonists and distinguishes agomelatine both to benzodiazepines (decreased release of 5-HT)^26,78,140^ and to SSRIs and SNRIs which elevate synaptic levels of 5-HT by blocking 5-HT reuptake sites on serotonergic terminals: increases are seen both acutely and upon long-term administration.^62,89^ Agomelatine may also be contrasted in this respect to buspirone, which decreases extracellular levels of 5-HT in corticolimbic territories by recruitment of 5-HT_1A_ autoreceptors on raphe cell bodies.^35,140^ In contrast to other classes of anxiolytic, then, agomelatine exerts its anxiolytic properties in the apparent absence of alterations in the release of 5-HT.

## Relationship of the anxiolytic properties of agomelatine to its antidepressant actions

Blockade of 5-HT_2C_ receptors and activation of MT receptors are the core mechanisms of action harnessed by agomelatine both in the treatment of GAD and, as amply discussed elsewhere, in the management of major depression.^22,23^ Clearly, then, the anxiolytic and antidepressant actions of agomelatine are fully compatible, and they are expressed over similar dose-ranges in patients with GAD and major depression, respectively. Under conditions of comorbid GAD and depression, antidepressant and anxiolytic properties may mutually reinforce each other. Interestingly, furthermore, relief of anxious states may help hinder the course to major depression.^141^ Although formal trials remain to be performed, these elements, combined with the ability of agomelatine to counteract the anxiety associated with chronic stress in rats (*vide supra*) suggest that it should relieve ‘mixed’ anxio-depressive conditions in patients.^7,22,48^

Noting interconnections between the anxiolytic and antidepressant actions of agomelatine is not, however, to contend that the same populations of 5-HT_2C_ and MT receptors and the same downstream substrates are implicated. Indeed, the above-discussed neural mechanisms engaged by agomelatine in the relief of anxious states are unlikely to mediate its impact on major depression. Conversely, pivotal to the antidepressant actions of agomelatine is its enhancement of the activity of dopaminergic and adrenergic pathways projecting to the FCX. This effect is unrelated to the anxiolytic properties of agomelatine, despite a possible role for 5-HT_2C_ receptors in the FCX – interconnected with the amygdala-BNST.^35,113,142,143^

## General discussion: open questions and perspectives

Finally, additional study should provide further insights into the mechanisms of action of agomelatine in the treatment of GAD.

First, at the cellular level, by analogy to 5-HT_2C_ receptor-Oxytocin receptor heterodimers (*vide supra*), a physical interaction between 5-HT_2C_ and MT_2_ receptors has been demonstrated both in cellular expression systems, as well as the hippocampus and cortex of rats.^144,145^ 5-HT_2C_-MT_2_ functional heterodimers possess ligand recognition and coupling properties that differ from the constituent monomers and dimers. Since agomelatine potently recognizes these heterodimers, it has been speculated that they may be involved in the clinical actions of agomelatine in depression. The same might be contended for GAD. However, while there is increasing evidence for the relevance of heteromeric G-protein-coupled receptor (GPCR) complexes to central nervous sytem (CNS) disorders,^146,147^ it is not yet known whether 5-HT_2C_-MT_2_ heterodimers are affected in the brain of GAD patients, nor whether their activity is altered under conditions of stress. Furthermore, ligands highly selective for 5-HT_2C-_MT_2_ heterodimers *versus* constituent monomers would be needed to rigorously evaluate their functional significance. Such agents are being sought but have not yet been described.^145^

*Second*, at the neurochemical and network level, it would be interesting to determine whether other neuromediators interlinked with 5-HT_2C_ receptors and known to influence anxious states, like cannabinoids and Neuropeptide Y, are involved in the actions of agomelatine.^148–150^ Furthermore, induction of brain-derived neurotrophic factor (and neurogenesis) in the hippocampus and FCX has been related to the antidepressant actions of agomelatine – and many other antidepressants – and it may be more generally involved in the response to stress and anxious states.^23,115,150–152^. Interestingly, 5-HT_2C_ receptor knockout mice reveal increased expression of brain-derived neurotrophic factor in the hippocampus.^153^ It would also be insightful to acquire a clearer picture of the neural structures where agomelatine exerts its actions, exploiting both animal models and human subjects. In addition to the amygdala-BNST, the hippocampus and the FCX (Figure 2), other structures warrant investigation such as the GAD-implicated habenula.^3^ In this MT receptor–rich structure,^123^ 5-HT_2C_ receptors play a role in the control of anxiety.^122,154,155^ 5-HT_2C_ receptors localized in the dorsal striatum also participate in the induction of anxious states.^118,156^ A final structure worth citing that possesses both MT receptors and 5-HT_2C_ receptors is the periaqueductal grey: this midbrain region is involved in the triggering of anxiety and has been identified as a site of action of 5-HT_2C_ antagonists.^6,122,157,158^ In addition to animal studies, clarification of neural circuits involved in the anxiolytic actions of agomelatine could be attempted in human subjects. This enterprise is however complicated – notwithstanding the sustained efforts of many laboratories – by the lack of specific positron emission tomography (PET)-imaging ligands.^159^ An alternative approach, highlighted by work in rodents, would be fMRI and electroencephalographic strategies for exploring circuits involved in the relief of GAD by agomelatine in comparison to other classes of agent.^85,108,112,160^

Third, the anxiolytic effects of agomelatine are expressed principally *via* 5-HT_2C_ receptors and ‘directly’ in interaction with corticolimbic and other subcortical circuits controlling anxious states. Nonetheless, in a clinical context, a beneficial influence of agomelatine on sleep patterns quality and circadian rhythms would be helpful in the relief of GAD and the improvement of quality of life. The influence of agomelatine upon sleep onset and rhythms is mainly melatonergic (MT receptor stimulation) in nature,^161–163^ but a contribution of 5-HT_2C_ receptor blockade should not be neglected.^85,110,111,164^ In fact, blockade of 5-HT_2C_ receptors likely contributes to the short-term improvement by antidepressants like trazodone and mirtazapine of sleep, although their sedative properties – due to histamine H_1_ antagonism – become problematic in some patients.^48,165,166^ Conversely, an influence upon sleep of agomelatine (which possesses neither affinity for H_1_ receptors nor marked sedative properties) does not play a major role in its antidepressant properties.^22,110,165,166^ Hence, to answer the question of whether – and by which mechanisms – a positive influence of agomelatine upon sleep and daily cycles putatively contributes to its relief of GAD, dedicated studies in patients will be required.^22,28,165,167^

Fourth, since agomelatine has only been evaluated in adult populations for the relief of GAD, it would be of interest to examine its potentially beneficial influence on GAD in specific populations like the young, including children and adolescents. Finally, in view of positive results in tests of social interaction in rodents^48^ and the social dimension of GAD,^164^ clinical studies of Social Anxiety Disorder and specific types of social phobia would be of interest

Finally, agomelatine is currently the only clinically authorized compound to possess a co-joint 5-HT_2C_ receptor antagonist plus MT_1_/MT_2_ agonist profile. Nonetheless, at least one new agent (GW117) with a comparable binding profile has recently been documented.^168^ Furthermore, it would be interesting to explore complementary ‘multi-target’ classes of agent articulated around 5-HT_2C_ receptor antagonist and/or MT agonist profiles for their potential utility in the improved treatment of GAD and other classes of anxiety disorder.

## Concluding comments

In conclusion, agomelatine expresses its therapeutic efficacy in GAD principally *via* its antagonist properties at 5-HT_2C_ receptors with MT_1_/MT_2_ agonism providing complementary anxiolytic properties. Its actions at these receptors are distributed across several brain structures like the hippocampus, amygdala-BNST, SCN, and TRN, and they are expressed in interaction with a suite of neurotransmitters and neuropeptides like glutamate, CRF, and vasopressin, but the precise underlying substrates await further clarification. Agomelatine displays a novel and fundamentally different mechanism of anxiolytic action as compared to all other classes of medication used to treat GAD, accounting for its clinical efficacy in the relative absence of deleterious actions.

## Supplemental Material

sj-docx-1-tpp-10.1177_20451253221105128 – Supplemental material for Agomelatine for the treatment of generalized anxiety disorder: focus on its distinctive mechanism of action Click here for additional data file. Supplemental material, sj-docx-1-tpp-10.1177_20451253221105128 for Agomelatine for the treatment of generalized anxiety disorder: focus on its distinctive mechanism of action by Mark J. Millan in Therapeutic Advances in Psychopharmacology

sj-pdf-2-tpp-10.1177_20451253221105128 – Supplemental material for Agomelatine for the treatment of generalized anxiety disorder: focus on its distinctive mechanism of action Click here for additional data file. Supplemental material, sj-pdf-2-tpp-10.1177_20451253221105128 for Agomelatine for the treatment of generalized anxiety disorder: focus on its distinctive mechanism of action by Mark J. Millan in Therapeutic Advances in Psychopharmacology
