# Supplementary material for: Agomelatine for the treatment of generalized anxiety disorder: focus on its distinctive mechanism of action
Source: Ther Adv Psychopharmacol. 2022 Jun 30;12:20451253221105128. doi: 10.1177/20451253221105128 (PMC9251978; doi:10.1177/20451253221105128)
Supplement: sj-docx-1-tpp-10.1177_20451253221105128 – Supplemental material for Agomelatine for the treatment of generalized anxiety disorder: focus on its distinctive mechanism of action [file sj-docx-1-tpp-10.1177_20451253221105128.docx]

# How Agomelatine Helps in the Treatment of Anxiety Disorders

**Introduction:**

- Anxiety disorders have a significant negative impact on quality of life.
- The most common type of anxiety disorder, called generalized anxiety disorder (GAD), is associated with nervousness and excessive worry
- These symptoms can lead to additional symptoms like tiredness, sleeplessness, irritability, and poor attention.
- GAD is generally treated through either cognitive-behavioral therapy or medication. However, widely used drugs like benzodiazepines and serotonin reuptake inhibitors have adverse effects.
- Agomelatine, a well-established anti-depressant drug, has shown anxiety-lowering (“anxiolytic”) properties in rats and has been shown to effectively treat GAD with minimal side effects.
- However, exactly how it acts on the brain to manage GAD is not yet clear.
- Thus, this review aims to shed light on agomelatine’s mechanism of action in treating GAD.

**Methods**

- The authors reviewed studies on how agomelatine treats anxiety in animals.
- They also looked at clinical studies on the effects of agomelatine in people with GAD.

**Results**

- The study showed that agomelatine “blocks” a receptor in nerve cells that plays a role in causing anxiety, called the 5-HT_2C_ receptor.
- Blocking this receptor, especially in specific brain regions such as nerve cells of the amygdala, bed nucleus of stria terminalis, and hippocampus, produced the anxiety reduction seen during agomelatine treatment.
- Agomelatine also activates the melatonin (MT) receptor, which is known to keep anxiety in check, promote sleep, and maintain the sleep cycle.
- Agomelatine should thus tackle sleep disturbances commonly seen in patients with GAD.
- Beyond 5-HT_2C_ and MT receptors, signaling molecules in nerve cells that are known to be involved in anxiety disorders (called “neurotransmitters” and “neuropeptides”) are also affected by agomelatine.

**Conclusion**

- Agomelatine's anxiolytic effects are caused by mechanisms that are distinct from those of other medications currently used to treat GAD.
- This explains its therapeutic success and minimal adverse side effects.

# Title of the paper: Agomelatine for the treatment of Generalized Anxiety Disorder (GAD): focus on its distinctive mechanism of action

# Authors: Mark J Millan

# Corresponding author e-mail: Mark.john.millan@gmail.com
