# Supplementary material for: Agomelatine for the treatment of generalized anxiety disorder: focus on its distinctive mechanism of action
Source: Ther Adv Psychopharmacol. 2022 Jun 30;12:20451253221105128. doi: 10.1177/20451253221105128 (PMC9251978; doi:10.1177/20451253221105128)
Supplement: sj-pdf-2-tpp-10.1177_20451253221105128 – Supplemental material for Agomelatine for the treatment of generalized anxiety disorder: focus on its distinctive mechanism of action [file sj-pdf-2-tpp-10.1177_20451253221105128.pdf]

How Does Agomelatine Aid in Managing Generalized Anxiety Disorder?

Generalized anxiety disorder (GAD) is one of the most commonly diagnosed forms of anxiety disorders

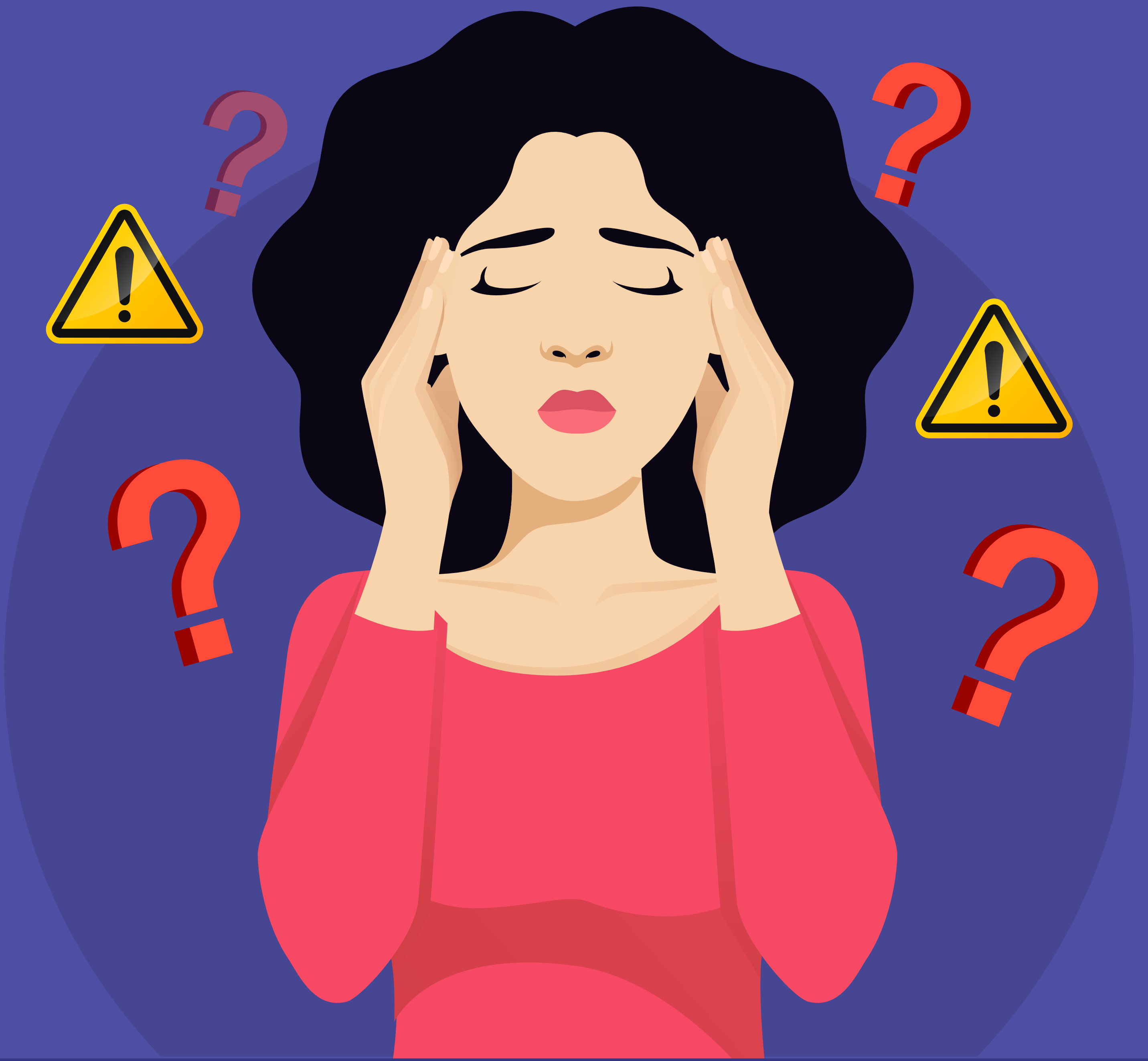

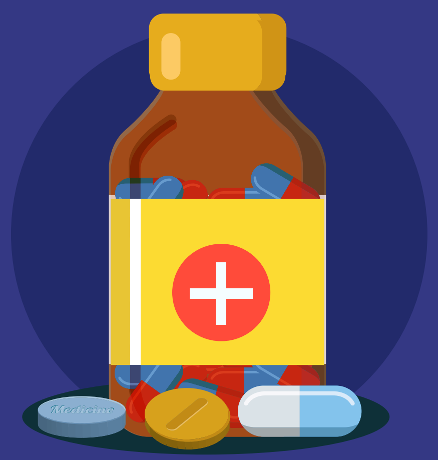

Among antidepressants, agomelatine has proven to be effective in the treatment of GAD, even in severely ill patients, as well as in preventing relapses

Extensive evidence supports a role of 5-HT_{2C} receptors in the induction of anxious states, with a complementary role of melatonin (MT) receptors

Mechanism of action

Agomelatine is unique in both blocking serotonin 5-HT_{2C} receptors and activating MT receptors, without recognizing monoamine transporters or GABA receptors

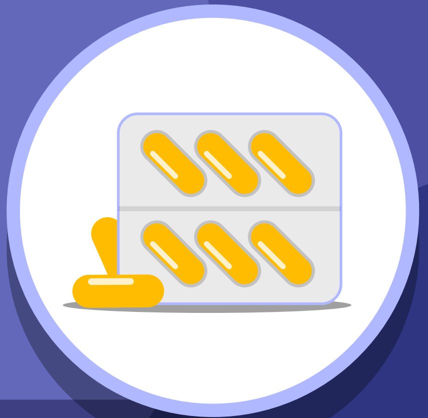

By inhibiting excess activity at 5-HT_{2C} receptors, agomelatine counters anxious states

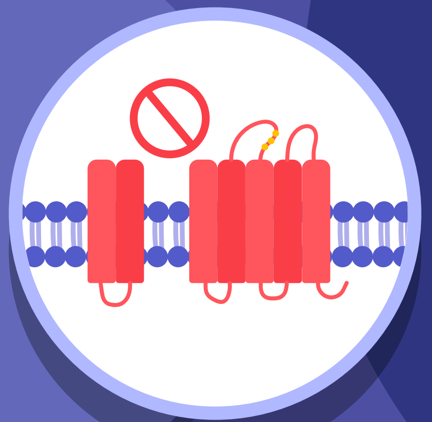

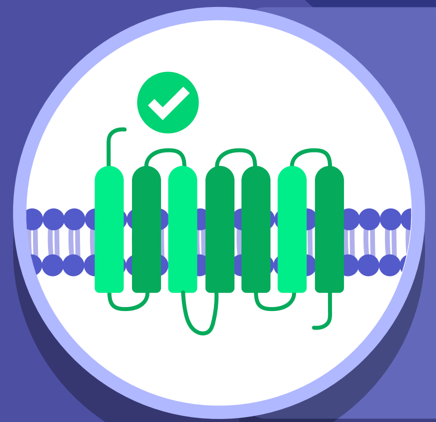

Agomelatine also activates MT receptors, which strengthens the alleviation of anxiety

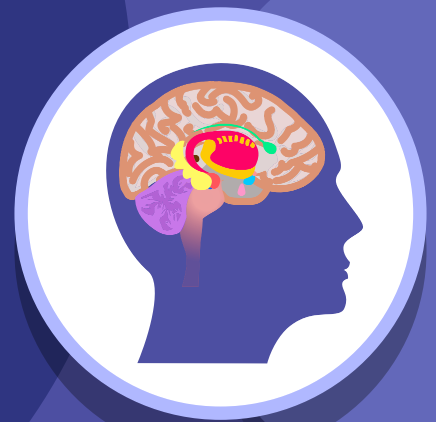

These anxiolytic actions are expressed at diverse brain structures, like the amygdala and the hippocampus

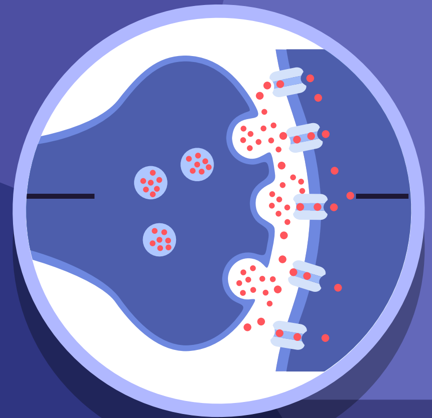

Anxiolytic effects of agomelatine involve the modulation of neurotransmitters and neuropeptides, like glutamate and oxytocin

The therapeutic success of agomelatine can be attributed to its mechanism of anxiolytic action, which is distinct from those of other agents currently employed for the management of GAD

Agomelatine for the treatment of generalized anxiety disorder (GAD): focus on its distinctive mechanism of action
MILLAN MARK J

Therapeutic Advances in Psychopharmacology
journals.sagepub.com/home/tpp

SAGE Publishing
